# Making glycoproteins a little bit sweeter with *PDB-REDO*

**DOI:** 10.1107/S2053230X18004016

**Published:** 2018-07-26

**Authors:** Bart van Beusekom, Thomas Lütteke, Robbie P. Joosten

**Affiliations:** a Division of Biochemistry, Netherlands Cancer Insitute, Plesmanlaan 121, 1066 CX Amsterdam, The Netherlands; b Institute of Veterinary Physiology and Biochemistry, Justus-Liebig-University Giessen, Frankfurter Strasse 100, 35392 Giessen, Germany

**Keywords:** glycoproteins, *PDB-REDO*, *pdb-care*, validation, carbohydrates

## Abstract

Glycosylation is one of the most common forms of protein post-translational modification, but is also the most complex. Dealing with glycoproteins in structure model building, refinement, validation and PDB deposition is more error-prone than dealing with nonglycosylated proteins owing to limitations of the experimental data and available software tools. Also, experimentalists are typically less experienced in dealing with carbohydrate residues than with amino-acid residues. The results of the reannotation and re-refinement by *PDB-REDO* of 8114 glycoprotein structure models from the Protein Data Bank are analyzed. The positive aspects of 3620 reannotations and subsequent refinement, as well as the remaining challenges to obtaining consistently high-quality carbohydrate models, are discussed.

## Abstract 2

The results and challenges of carbohydrate handling in the current PDB-REDO databank are discussed.

## 1. Introduction   

Structural biology provides us with insight into the molecular mechanisms of life (Brändén & Tooze, 1998 ▸). These mechanisms are mostly viewed from the perspective of proteins because of their many roles as, for instance, chemical converters, structural building blocks and signal processors, but proteins do not work alone. Their interactions with each other and with nucleic acids, small molecules, ions and even water molecules form the basis of very complex systems and interaction networks. These systems and networks are regulated to deal with changes in the functional requirements, for example owing to environmental factors, at any given time. Changes to the covalent structure of the protein, *i.e.* post-translational modifications, are probably the most well known form of regulation.

Glycosylation is not only one of the most common but also one of the most complex forms of co-translational and post-translational modifications of proteins. Glycan chains are involved in protein folding *via* the calnexin/calreticulin cycle (Caramelo & Parodi, 2008 ▸), serve as molecular markers, influence protein stability and influence the function of proteins (Varki, 2017 ▸). While the core structure of N-glycans is highly conserved, a large variety of different structures can be found outside the N-glycan core as well as in O-glycans (Varki, 2017 ▸). This variety of glycans allows the fine-tuning of protein functionality (Varki, 2017 ▸; Ohtsubo *et al.*, 2011 ▸; Nigrovic, 2013 ▸). Glycans play important roles in cell signalling and trafficking (Furukawa *et al.*, 2012 ▸; Kiermaier *et al.*, 2016 ▸) as well as in infections, inflammation and the immune response (Varki, 2017 ▸; Schnaar, 2015 ▸; Frabutt *et al.*, 2018 ▸; Tytgat & Vos, 2016 ▸), where glycans are specifically recognized by glycan-binding proteins, the lectins, or by antibodies. In all of these events small differences in glycan chains, such as different stereochemistries of single C atoms or different linkage positions, can have large effects on the biological function. For example, the carbohydrate-recognition domain of influenza virus haemagglutinin is specific for a particular linkage between a terminal sialic acid residue and the vicinal galact­ose: avian influenza virus haemagglutinin predominantly binds to (2–3)-linked sialic acid, whereas haemagglutinin from human influenza virus preferably recognizes (2–6)-linked sialic acid (Stevens *et al.*, 2006 ▸; Yang *et al.*, 2015 ▸).

The three-dimensional structures of glycoproteins and protein–carbohydrate complexes in the Protein Data Bank (PDB; Bernstein *et al.*, 1977 ▸) are a valuable resource for understanding the effects of glycosylation on proteins. Additionally, they provide a detailed molecular view of the recognition of glycans by proteins. Unfortunately, however, the glycan parts of these structures often feature many flaws ranging from minor irregularities to gross errors (Lütteke *et al.*, 2004 ▸; Crispin *et al.*, 2007 ▸; Agirre, Davies *et al.*, 2015 ▸). This problem has multiple causal factors: the median data resolution of glycoproteins (2.4 Å) is somewhat lower than that of PDB entries in general (2.0 Å); most software tools for structural biology are protein-centric and do not deal with carbohydrates as well as they deal with proteins; most crystallo­graphers are less well trained in dealing with carbohydrates; glycosylation sites on a protein may not be part of the research question for which a structure is solved (that is, little effort is spent on these); validation of carbohydrates is not part of the standard structure-elucidation process; and the deposition and annotation process of the wwPDB (Young *et al.*, 2017 ▸) is not focused on carbohydrates. All of these factors are understandable given the limited resources available to the parties involved, but there nevertheless is a drive to improve the quality of carbohydrates in available structure models (Read *et al.*, 2011 ▸).

The *PDB-REDO* pipeline for ‘constructive validation’ (Joosten *et al.*, 2012 ▸) of crystallographic structure models could be of service to crystallographers and other structural biologists for improving the quality of glycoprotein structure models but, like so many tools, *PDB-REDO* is protein-centric. It does, however, have a set of features for improving carbohydrates based on *pdb-care* (Lütteke & von der Lieth, 2004 ▸; Joosten & Lütteke, 2017 ▸). Because the *PDB-REDO* pipeline is fully automated, it treats models consistently. This, however, is only completely true within one version of the pipeline. Over the years, the PDB-REDO databank (Joosten *et al.*, 2009 ▸) has developed a considerable range of versions of software in the pipeline, which makes it hard to reliably analyze the performance of *PDB-REDO* with respect to carbohydrates. We recently replaced the PDB-REDO databank completely, which brought everything to a single version (van Beusekom *et al.*, 2018 ▸). Here, we use this opportunity to see where PDB-REDO stands in terms of the various aspects of carbohydrate structure model quality and what more can be improved in future incarnations of the PDB-REDO project.

## 2. Methods   

### 2.1. Data-set selection   

We analyzed all of the entries in the recently updated PDB-REDO databank (van Beusekom *et al.*, 2018 ▸) in which any of the monosaccharides β-d-GlcpNAc, α-d-GlcpNAc, α-d-Man*p*, β-d-Man*p*, β-d-Glc*p*, α-d-Glc*p*, β-d-Ga*p*, α-d-Gal*p*, α-l-Fuc*p* or β-l-Fuc*p* were present and annotated as separate residues. For example, maltose is only analyzed if it is annotated as two covalently bound glucose residues rather than as a single maltose residue. To stress this distinction, we will refer to the monosaccharides studied in this work by their PDB Chemical Component Dictionary (Westbrook *et al.*, 2015 ▸) residue names: NAG, NDG, MAN, BMA, BGC, GLC, GAL, GLA, FUC and FUL, respectively. The data set in this study consisted of 8114 PDB-REDO entries (out of a total of 111 130 entries) and their PDB counterparts.

### 2.2. Changes of carbohydrate annotation   

The *PDB-REDO* program *stripper* (Joosten *et al.*, 2012 ▸) changes the annotations of carbohydrates in PDB-format structure models to streamline restraint generation for refinement in *REFMAC* (Murshudov *et al.*, 2011 ▸). This re­annotation, based on the output of *pdb-care* (Lütteke & von der Lieth, 2004 ▸), has been described previously (Joosten & Lütteke, 2017 ▸). Briefly, changes brought on by the reannotation of carbohydrates can include residue renaming, deletion of superfluously modelled leaving atoms (*i.e.* the O1 atoms of residues), removal and addition of LINK records describing covalent linkages between residues, standardization of glycosidic linkages to describe O1 as the leaving atom and explicit annotation of the expected linkage type [for example β(1–4) between two NAG residues in an N-linked glycan]. For the selected data set, the final PDB-REDO model and the equivalent PDB entry were compared to find all annotation changes.

### 2.3. Model-quality analysis   

The quality of carbohydrates in structure models was analyzed using several tools. We measured the fit to the experimental data using *EDSTATS* (Tickle, 2012 ▸), checked the residue conformation with *Privateer* (Agirre, Iglesias-Fernández *et al.*, 2015 ▸), computed the chiral volume of the anomeric centre atom C1 as described by Evans (2007 ▸) and validated the torsion of glycosidic linkages with an updated version of *CARP* (Lütteke *et al.*, 2005 ▸). This new implementation of *CARP* expresses the linkage-specific torsion-angle set (φ, ψ) as a knowledge-based potential with respect to high-quality distributions of torsion angles from GlycoMapsDB (Frank *et al.*, 2007 ▸). For consistency, the final PDB-REDO model and the model after reannotation, but before any refinement, were compared. That is, the residue names and LINK records that are used in *PDB-REDO* are used for validation against residue-specific or linkage-specific geometric parameters.

### 2.4. Carbohydrate hydrogen bonds and homology   

We recently showed that using homology-based hydrogen-bond restraints in refinement can help to improve protein structure models at medium and low resolution (van Beusekom *et al.*, 2018 ▸). Carbohydrates have a large set of hydrogen-bond donors and acceptors, but the relative quality of carbohydrate structures is poor. Here, we investigate whether there are enough suitable hydrogen bonds in glycoproteins to create hydrogen-bond restraints for carbohydrates.

The program *Sweethy* was developed to analyze hydrogen bonds that involve carbohydrate residues and to find their homologous equivalents. The program was based on the algorithms in *HODER* (van Beusekom *et al.*, 2018 ▸) with extensions specifically for carbohydrates. Upon reading an input PDB file, carbohydrate moieties were identified using their annotation as ‘pyranose’ in the CCP4 monomer library (Vagin *et al.*, 2004 ▸). The restraint files of the carbohydrate residues present were parsed to identify the atoms and their bonds with their bond orders. From these, it was determined which atoms are hydrogen-bond donors and/or acceptors. Hydrogen bonds between two carbohydrates and between the protein and a carbohydrate were detected based on the same geometric criteria that we used before (van Beusekom *et al.*, 2018 ▸). In the current implementation, homologous protein–carbohydrate hydrogen bonds are only identified for N-linked glycosylation trees as these can be matched reliably. The protein residue that forms a protein–carbohydrate hydrogen bond is identified, followed by identification of the equivalent residue in homologues. Also, the residue homologous to the asparagine from which the glycosylation tree sprouts is identified. From this residue, the homologous carbohydrate residue is found by looking for a carbohydrate moiety in the same relative position in the glycosylation tree. It should be noted that the given carbohydrate residue type is purposely ignored because these data are not completely reliable.

To assess whether general hydrogen-bond restraints for carbohydrates are a viable option, we analyzed hydrogen bonds in the glycoproteins in our data set. Additionally, we looked for conserved hydrogen bonds in homologous structures based on which homology-based hydrogen-bond restraints can be generated.

## 3. Results and discussion   

### 3.1. Annotation changes   

The structure models in the data set were all treated by *PDB-REDO* v.7.00 or higher, which means that they were all checked for solvable annotation errors involving carbo­hydrates. Of the 66 137 carbohydrate residues considered, 1732 were renamed (Table 1 ▸). This is a conservative estimate of the total number or residues that need renaming, as only cases where the identity of residue can be reliably assigned based on prior knowledge (for example, as is the case for the core residues of N-linked glycans) are handled automatically. In particular, a large percentage of all NDG and FUL residues (63 and 77%, respectively) were renamed because these are frequently erroneously added to N-linked glycans at positions where NAG and FUC residues should have been added. Other cases in which the model coordinates of a particular carbohydrate residue are in conflict with the residue type, but no prior knowledge on which type to expect is available, are also detected by *pdb-care*. The suggested residue-renaming actions, however, are not applied by *stripper* as these are associated with a somewhat higher false-positive rate, which makes them unsuited for fully unsupervised reannotation in the context of *PDB-REDO*.

**Figure 1:**
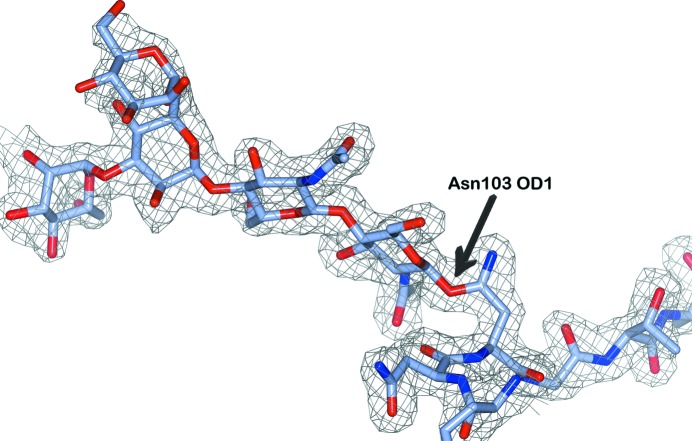
An example of incorrect modelling of N-glycosylation in the structure of the laccase McoG (PDB entry 5lm8; Ferraroni *et al.*, 2017 ▸). The first NAG residue is attached to the O^δ^ atom (OD1 in PDB nomenclature) of Asn103, rather than to the N^δ^ atom. The clear electron density (2*mF*_o_ − *DF*_c_ at 1.2σ; clipped around the residues for clarity) and the fact that the neighbouring N-glycosylation on Asn60 is correct suggest that this is a simple oversight. Errors of this type are automatically corrected in *PDB-REDO* by swapping the names of the O^δ^ and N^δ^ atoms and updating the linkage description accordingly. This figure was produced using *CCP*4*mg* (McNicholas *et al.*, 2011 ▸).

In addition to residue renaming, there were many annotation modifications that involved LINK records: 286 LINKs were removed, 885 were added and 717 were replaced. A special case occurs when the first NAG residue of an N-linked glycan is linked to the O^δ^ atom of the asparagine side chain. These cases are fortunately quite rare (21 in total; for a recent example, see Fig. 1 ▸). They are treated by flipping the asparagine side chain by 180° and modifying the LINK record to bind the NAG residue from the N^δ^ atom.

**Figure 2:**
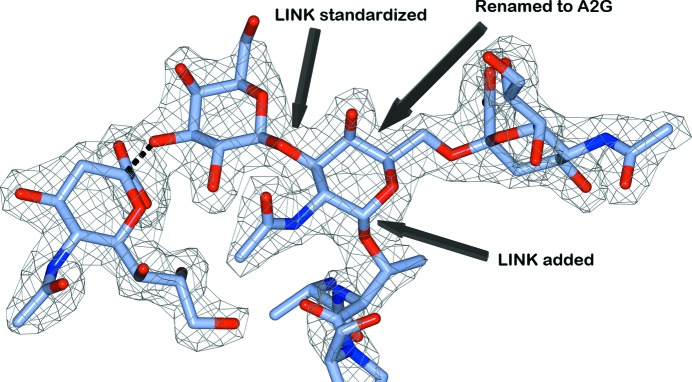
The sialoglycopeptide from glycophorin A (chain *D* of PDB entry 2cwg; Wright & Jaeger, 1993 ▸) required multiple reannotation events: a LINK record was added to connect the threonine side chain to the central carbohydrate residue, the central residue NDG was renamed A2G (α-d-Gal*p*NAc) and a LINK record between GAL and A2G was rewritten to correctly describe the leaving atom O1. The dotted line represents a glycosidic linkage that was already present in the PDB entry but is not displayed by *CCP*4*mg*.

It is not uncommon for several reannotation events to be required in a single structure model. An example is found in the structure of wheat germ agglutinin in complex with a glycosylated peptide from glycophorin A (Wright & Jaeger, 1993 ▸), which required three types of reannotation: LINK standardization, LINK addition and residue renaming (Fig. 2 ▸). In this structure model the atomic coordinates of the glycos­ylated peptide in chain *D* are of high quality, but without reannotation this model would deteriorate severely during refinement owing to incorrect restraints.

A case where the current reannotation in *PDB-REDO* is insufficient is found in the crystal structure of the carbo­hydrate-recognition domain of dectin-2 in complex with a Man_9_GlcNAc_2_ oligosaccharide (Feinberg *et al.*, 2017 ▸). In this crystal structure the oligosaccharide is in a crystallographic special position, which means that it overlaps with a symmetry-related copy of itself in the electron density. This was correctly modelled in the deposited model (PDB entry 5vyb), but the annotation from the PDB included LINK records that connected the mutually exclusive symmetry copies of the saccharide. This caused *pdb-care* to incorrectly interpret the model, which caused *stripper* to remove the wrong atoms; this in turn caused *REFMAC* to generate very incorrect restraints and thus distort the model significantly. When the offending LINK records were removed, all of the steps in the *PDB-REDO* pipeline worked as expected. A bug report was sent to the PDB annotators to ensure that the incorrect LINK records are removed permanently.

### 3.2. Model-quality indicators   

#### 3.2.1. Density fit   

The results from model analysis with *EDSTATS* and *Privateer* are summarized in Table 2 ▸. The subset of high-resolution cases (<1.8 Å) was also analyzed separately. We observe an overall improvement in fit to the electron density: the median real-space correlation coefficient (RSCC; Brändén & Jones, 1990 ▸) increased from 0.84 in the PDB to 0.87 for PDB-REDO. In the high-resolution subset a similar improvement is found, with the median RSCC increasing from 0.91 to 0.93. This improvement in RSCC is probably mostly caused by a general improvement of the models in PDB-REDO (Joosten *et al.*, 2012 ▸).

#### 3.2.2. Carbohydrate-ring conformations   

The θ angle that describes the carbohydrate-ring conformation (Cremer & Pople, 1975 ▸) should be close to 0° for the d-pyranoses and close to 180° for the l-pyranoses considered in this study if the residue is in the most favourable chair conformation. Indeed, for high-resolution carbohydrates we find that the angles in both PDB and PDB-REDO are around 6.5 and 175°, with small nominal changes for the better in PDB-REDO (Table 2 ▸). The quality of carbohydrate rings at high resolution is good in the majority of cases. The main difference is that in PDB-REDO the three-tier validation status given by *Privateer* (good/check/bad) is far less often ‘bad’: in only 137 cases *versus* 239 cases in the PDB. In PDB-REDO 9083 residues are considered good *versus* 8937 residues in the PDB.

**Figure 3:**
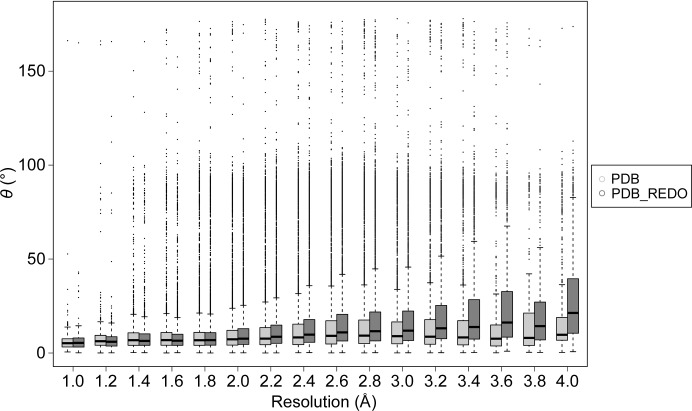
Distribution of θ angles in the PDB and in PDB-REDO. The data are displayed as box-and-whiskers plots with the whiskers extending to 1.5 times the interquartile range. In the PDB the θ angle that describes the carbohydrate-ring conformation increases gradually, while in PDB-REDO this effect is magnified.

If the medium- and low-resolution models are also taken into account we find that the median θ angles are somewhat worse overall in the PDB: 7.9 and 173°. In PDB-REDO the median θ angle deteriorates for the d-pyranoses to a median of 9.2°, but in l-pyranoses there is a minor improvement of 0.6°. The observation that θ angles deviate more from the target owing to the contribution of lower resolution cases is expected: it is a clear indication that the carbohydrate structure quality decreases with resolution. This effect is however much stronger in PDB-REDO than in the PDB (Fig. 3 ▸).

The most probable reason for this is a combination of how *PDB-REDO* deals with geometric restraint weighting and the way that geometric restraints for carbohydrates are defined in the CCP4 monomer library. *PDB-REDO* optimizes the overall geometric restraint weight with respect to the X-ray data to give the best fit to the data while still maintaining ‘reasonable’ model geometry. This reasonable geometry is defined as the bond-length and bond-angle root-mean-square *Z*-score being lower than 1. At low resolution this often means that *PDB-REDO* uses somewhat looser restraints than are commonly used for PDB entries (Joosten *et al.*, 2009 ▸). This improves the overall geometric quality of the protein part of structure models (van Beusekom *et al.*, 2018 ▸), but clearly not carbohydrates. The looseness of carbohydrate restraints can be traced back to the individual weights of geometric restraints in the CCP4 monomer library, which are defined by a tolerance or sigma value. For amino-acid residues these values are case-specific and are taken from the distribution of bond lengths and angles in high-resolution small-molecule structures (Engh & Huber, 1991 ▸); for carbohydrate residues these tolerance values are set to a relatively high, fixed value. This means that restraints for carbohydrates are relatively weak. With decreasing resolution, *i.e.* when restraints become more important, the relative weakness of carbohydrate restraints with respect to protein restraints becomes apparent and carbohydrate geometry starts to deviate more from ideal values.

A logical solution would be to update the tolerances for individual carbohydrate restraints based on data mining very high-quality structures. This is exactly what the program *AceDRG* does (Long *et al.*, 2017 ▸). We are working with the *AceDRG* developers to update the carbohydrate restraint files in the CCP4 monomer library.

Apart from bond lengths and angles, other restraints such as torsional restraints may be generated to keep the ring conformation of high quality. Given the current results, it is not obvious that these are strictly needed. This needs to be tested with a new set of restraints. As previously suggested, restraints on torsion angles can be used to force distorted carbohydrate rings into the most favourable chair conformation (Agirre, Davies *et al.*, 2015 ▸). This should of course only be performed if no other conformation can be expected based on the structural context (for example when the residue is in a chemical transition state). The θ angle itself, however, is not a suitable value to restrain, especially because it also represents an independent validation criterion, much like Ramachandran plot angles for proteins (Ramachandran *et al.*, 1963 ▸). Restraining against validation metrics makes them less usable since the data will become biased.

In some cases, large changes in ring conformation occur: if a carbohydrate with the wrong residue name was refined with tight restraints the conformation will be quite good; if the residue name is now changed to match the biological residue identity (as we do in many cases) the next refinement will force one or more chiral inversions of the ring atoms; if this inversion is not completed (mostly because the bond-angle restraints work against the chiral volume restraints) the residue ends up in a distorted conformation.

**Figure 4:**
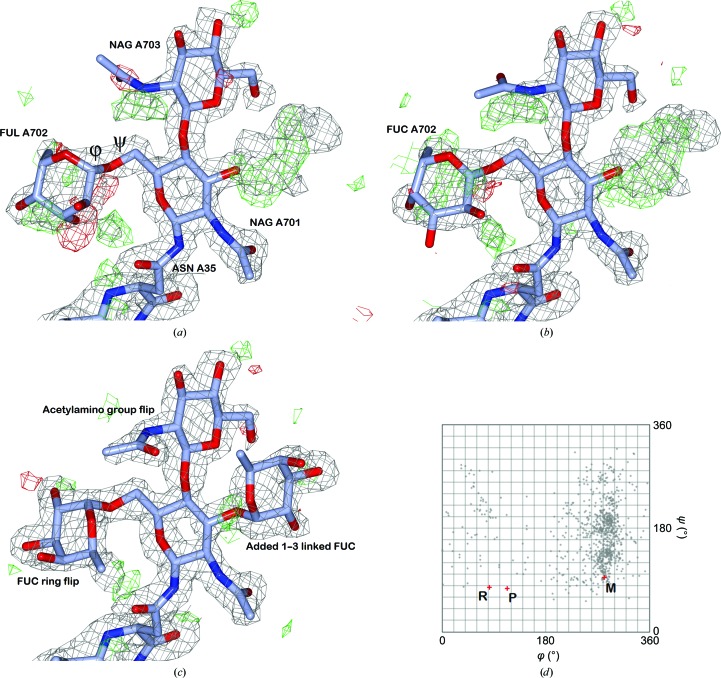
An N-linked glycan attached to Asn35 of human Toll-like receptor 4 (PDB entry 2z62; Kim *et al.*, 2007 ▸). Electron density is contoured at 1σ for the 2*mF*_o_ − *DF*_c_ map (grey) and 3σ for the *mF*_o_ − *DF*_c_ difference density map (green, positive; red, negative). (*a*) Model as found in the PDB with the (1–6)-linked fucose incorrectly modelled as FUL. The density near the O6 atom of asparagine-linked NAG is partially filled by four water molecules in a symmetry-related copy of the model (purposely not shown), which reduces the amount of positive difference density. (*b*) PDB-REDO model and map. The fucose residue is renamed FUC. Subsequent refinement improves the fit to the electron density, but distorts the ring conformation and flattens the hand of the C1 atom. (*c*) Manually rebuilt model after refinement by *PDB-REDO*. The (1,6)-linked fucose is flipped to correctly fit the density, as is the acetylamino group of the second NAG residue. Together with adding a (1–3)-linked fucose, these corrections remove all strong difference density. (*d*) The *CARP* plot for (1–6)-linked fucose shows the distribution of FUC-(1–6)-NAG glycosidic linkage torsion angles in the PDB. The relevant bonds in the glycosidic linkage are marked in (*a*). The models are marked as follows: PDB, P; PDB-REDO, R; manually rebuilt, M. Only the manually rebuilt model has common glycosidic torsion angles.

To explore this, we looked at the θ-angle statistics of only those residues that were renamed (Supplementary Table S1). For these cases we see that the θ angles are indeed worse than in the general case both in the PDB and in PDB-REDO. For the d-pyranoses we see that the nominal effect of re-refinement in PDB-REDO is larger: high-resolution cases improve more and low-resolution cases deteriorate more in terms of median θ angle. For the fucose residues (FUC and FUL) we see strong deteriorations for the renamed cases. A reason for this could be that the residues have relatively poor electron density. With the caveat that there are only 16 high-resolution cases, we see that the median RSCC is indeed much lower than for the general high-resolution case: 0.74 in the PDB and 0.78 in PDB-REDO. Combining poor density with the fact that the renamed fucose residues are exclusively found at the tips of carbohydrate tree branches (*i.e.* attached to the first NAG residues of N-glycans) suggests that there is much more room for error when building these residues. An example is found in PDB entry 2z62 (Kim *et al.*, 2007 ▸), where the fucose residue that is renamed is fitted with its ring plane upside down (Fig. 4 ▸). Visual inspection of the other high-resolution cases showed seven cases that needed rebuilding (five with ring flips), two cases where the fucose was not well enough supported by the density and one unclear case. The five remaining cases had structurally favourable ring configurations both in the PDB and PDB-REDO models. Here only the fucose residues had to move somewhat to accommodate the correct glycosidic linkage.

#### 3.2.3. Anomeric centre geometry   

**Figure 5:**
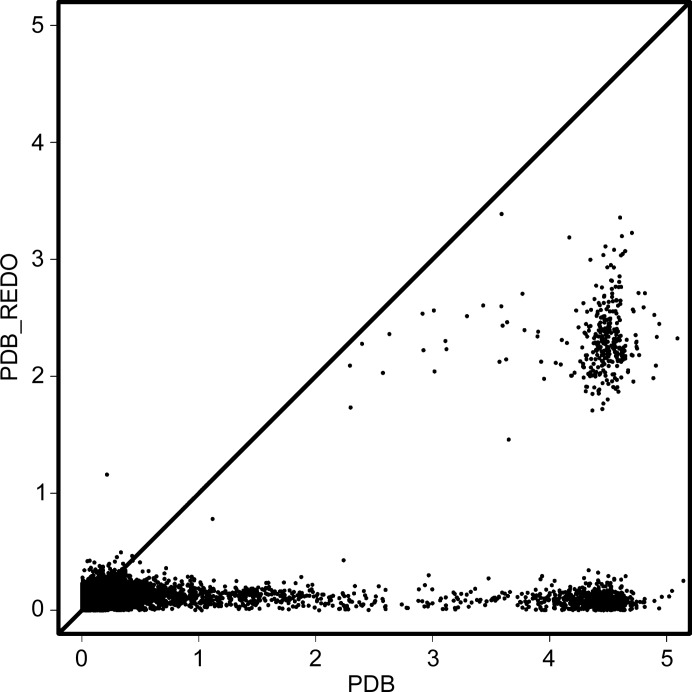
Distribution of chiral volume deviations (absolute values) of anomeric centre atoms C1 in the PDB and in PDB-REDO. The optimal value for chiral volume is either −2.22 (for an α-linkage) or +2.22 (for a β-linkage); corrections of chirality are therefore expected to change the chiral volume by about 4.5. A clear improvement can be observed for the vast majority of cases (all points below the diagonal). Three distinct clusters are observed in the plot: cases with the correct hand in both the PDB and PDB-REDO (bottom left), cases with the wrong hand in the PDB but the correct hand in PDB-REDO (bottom right) and cases where the hand was incorrect in the PDB and the anomeric centre ended up flat in PDB-REDO (middle right).

The distribution of the chiral volume deviations of the anomeric centre (C1 atom) in the PDB and in PDB-REDO was analyzed (Fig. 5 ▸). The renaming of residues and the explicit setting of anomer-specific restraints for glycosidic linkages can cause large initial deviations from the expected chiral volume. If the hand is completely inverted [for example when the model shows a β(1–2) linkage between two MAN residues] the distortion is between 4 and 5. Distortions of between 2 and 3 show that the anomeric centre is nearly flat. Lower distortions indicate that the hand of the anomeric centre is correct. We see a clear reduction of outliers after *PDB-REDO* even when the hand of the anomeric centre is completely opposite. Not all issues are resolved: some anomeric centres end up rather flat after *PDB-REDO*.

#### 3.2.4. Glycosidic linkage conformation   

**Figure 6:**
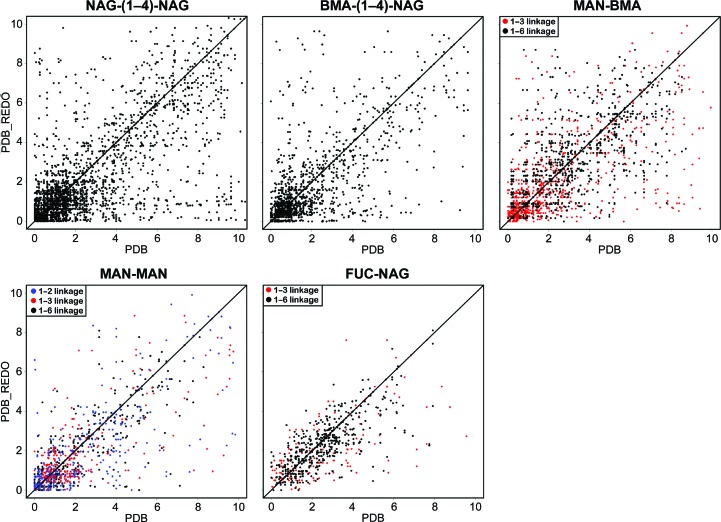
Comparison of knowledge-based potentials for glycosidic linkages commonly found in N-glycans in the PDB and in PDB-REDO. Potentials are calculated by *CARP* (Lütteke *et al.*, 2005 ▸) and are given in arbitrary units; lower values are better.

Glycosidic linkages have two torsion angles (φ and ψ) which *CARP* uses as coordinates in a Ramachandran-like plot. The typical torsion-angle distribution depends on the type of glycosidic linkage in terms of partner residues, ring positions and anomers, which makes it difficult to analyze large sets of linkages. The new implementation of *CARP* expresses the linkage conformation as a knowledge-based potential to make the analysis of large data sets more convenient. The potentials in the PDB and in PDB-REDO for linkages commonly found in N-glycans were compared per linkage type (Fig. 6 ▸), as was the complete set of potentials for all linkages that are recognized by *CARP* (Supplementary Fig. S1). Points close to the origin of the plots indicate favourable conformations. These are seen most commonly for NAG-NAG and BMA-NAG β(1–4) linkages. This is likely to be the result of two factors that make modelling the residues involved relatively easy: the residues are close to the protein and have therefore relatively clear density (compared with the more remote carbohydrate residues) and the extended flat shape of NAG residues leaves little room for alternative interpretation of this electron density. The torsion angles in glycosidic linkages are not restrained in refinement, so unlike the anomeric centre geometry no strong improvements are expected. Indeed, only a subtle trend towards improvement is seen. Manual rebuilding, particularly when ring flips are involved, can cause large glycosidic linkage torsion improvement (Fig. 4 ▸).

### 3.3. Hydrogen bonds   

**Figure 7:**
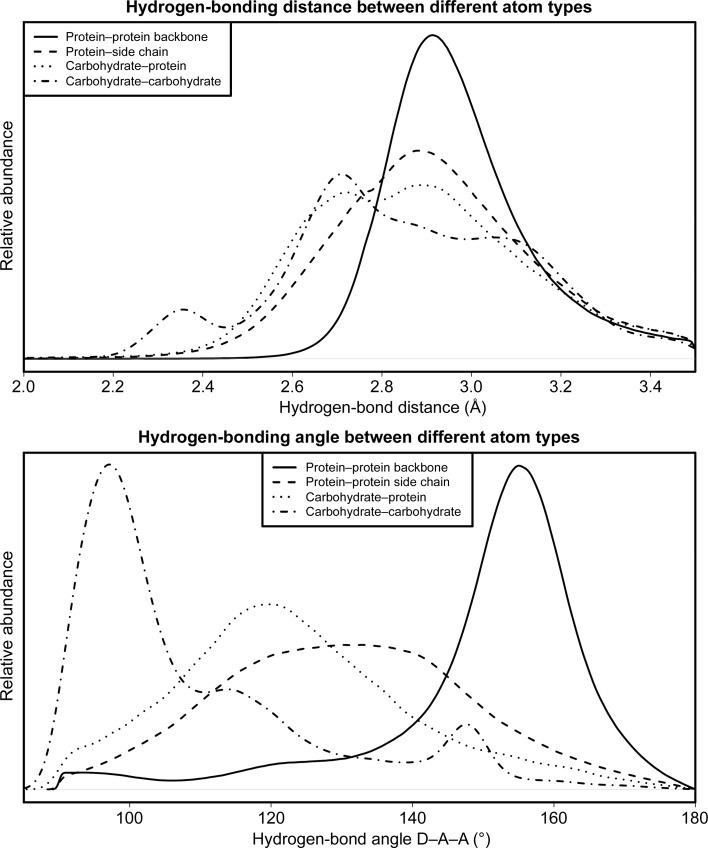
Distribution of hydrogen-bond parameters for different hydrogen-bonding types. The hydrogen-bond length distribution (top) for cases involving carbohydrates is much broader than for protein-only hydrogen bonds. The hydrogen-bond angle distributions (bottom) show that typical carbohydrate–carbohydrate hydrogen bonds have much sharper angles, indicating that they are relatively weak and not suited for generating hydrogen-bond restraints.

We detected 17 834 hydrogen bonds between two carbohydrate moieties in PDB-REDO (20 124 in the PDB) and 68 563 hydrogen bonds between carbohydrates and amino acids in PDB-REDO (66 499 in the PDB). The carbohydrate–protein hydrogen bonds, both in the PDB and in PDB-REDO, are of higher average quality than carbohydrate–carbohydrate hydrogen bonds: the angles are much better (*i.e.* closer to the ideal angle of 180°) for carbohydrate–protein hydrogen bonds and the hydrogen donor and acceptor distances form a narrower distribution (Fig. 7 ▸).

The methodology for generating protein–carbohydrate hydrogen-bond restraints was implemented in *Sweethy* as in *HODER* (van Beusekom *et al.*, 2018 ▸). Unlike *HODER*, *Sweethy* only creates restraints for which a homology-based target could be determined. General target restraints are not created because protein–carbohydrate hydrogen bonds have a very specific molecular context, which is seen in their wide distribution of distances and angles.

We investigated the extent to which homologous hydrogen-bond restraints could be generated. Therefore, we filtered the set of hydrogen bonds found before to keep only those which had at least five available homologues (which is the lower limit for homology-based restraint generation) and to keep only those hydrogen bonds which are found both in the PDB and the PDB-REDO structure (to ensure reliability of the hydrogen bond). This gave us a total of 6931 hydrogen bonds that may be restrained (Supplementary Fig. S2). However, we noted that the equivalent atoms in homologues were often not involved in a hydrogen bond: only in 2013 cases was the hydrogen bond fully conserved over all homologues. 2060 hydrogen bonds were conserved 50% of the time or less. The expected conservation of hydrogen bonds in homologues forms the basis of our earlier work on protein hydrogen-bond restraints (van Beusekom *et al.*, 2018 ▸). Protein–carbohydrate hydrogen bonds, however, are poorly conserved. Therefore, we concluded that protein–carbohydrate hydrogen bonds are not reliable enough to restrain, even based on homologous data.

### 3.4. Towards better carbohydrate models   

Structural biologists, the wwPDB and the developers of macromolecular crystallography and validation tools have collectively worked to produce ever better structure models, and this is also the case for the carbohydrate part of structure models. Since the end of the last decade there has been a steady improvement in the carbohydrate-ring conformations of new PDB entries (Supplementary Fig. S3). This coincides with the second wave of model-validation development that also resulted in the establishment of the PDB’s Validation Task Forces (Read *et al.*, 2011 ▸).

The results here show that there are a substantial number of cases of carbohydrate residues in the PDB that can be improved. The reannotation of residues and linkages in N-glycans removes many misrepresentations of known biology and is an important step to aid downstream studies of the structure–function relationships of N-glycosylation sites. At the same time, we know that there are other cases that cannot be corrected automatically and thus require manual curation. This is a huge undertaking that is beyond the scope of *PDB-REDO* and can only be performed by the wwPDB with a lot of help from the original depositors. The new policy of the PDB that allows depositors to update their models without having to obsolete them will hopefully encourage depositors to correct annotations if they notice them.

The combination of reannotation and refinement in *PDB-REDO* can correct certain errors and improves the handedness of anomeric centres. At the moment, ring conformations are improved only at high resolution; at low resolution ring conformations tend to become worse with the current set of restraints. There are clear cases where reannotation and refinement are insufficient to reach high-quality results in which both the annotation and the conformation of the carbohydrates is correct. In these cases, rebuilding of the carbohydrates is needed (Fig. 4 ▸ *c*). Thanks to large improvements in carbohydrate-building tools, most notably in *Coot* (Emsley *et al.*, 2010 ▸), this has now become relatively straightforward to perform interactively. In the context of *PDB-REDO*, however, this process should be achieved in a fully automated and unsupervised manner. This level of automation is not yet available, although steady progress in this direction is being made (Agirre *et al.*, 2017 ▸). Overall, glycoprotein structure is not yet optimal, but the improvements discussed here together with the many other developments in the field are moving structural quality in the right direction.

## Supplementary Material

Supplementary Table and Figures.. DOI: 10.1107/S2053230X18004016/va5010sup1.pdf

## Figures and Tables

**Table 1:** Carbohydrate-renaming events

Residue name		No. of renaming events	
PDB	PDB-REDO	Count	Percentage
NDG	NAG	552	63
MAN	BMA	553	6.6
BMA	MAN	303	6.9
FUL	FUC	287	77
Other	Other	37	ND

**Table 2:** The quality of carbohydrate residues in the PDB and PDB-REDO databanks Only data for the most prevalent carbohydrate residues (NAG, NDG, MAN, BMA, BGC, GLC, GAL, GLA, FUC and FUL) are used.

	Median θ angle† ‡ (°)	Median RSCC§	Validation status† (good/check/bad)
PDB, <1.8 Å	6.7 (174.9)	0.91	8937/590/239
PDB-REDO, <1.8 Å	6.4 (175.3)	0.93	9083/543/137
PDB, all	7.9 (172.7)	0.84	50931/7627/2512
PDB-REDO, all	9.2 (173.3)	0.87	49052/9784/2213

† As reported by *Privateer*.

‡ Values for FUC and FUL are given in parentheses.

§ As reported by *EDSTATS*.
