# Supplementary material for: Making glycoproteins a little bit sweeter with PDB-REDO
Source: Acta Crystallogr F Struct Biol Commun. 2018 Jul 26;74(Pt 8):463–72. doi: 10.1107/S2053230X18004016 (PMC6096482; doi:10.1107/S2053230X18004016)
Supplement: Supplementary file 1 [file f-74-00463-sup1.pdf]

STRUCTURAL BIOLOGY COMMUNICATIONS

Volume 74 (2018)

Supporting information for article:

Making glycoproteins a little bit sweeter with *PDB_REDO*

Bart van Beusekom, Thomas Lütteke and Robbie P. Joosten

Table S1 Median θ angles for carbohydrate residues[a] renamed by PDB-REDO

	Median θ angle (°) [b, c]	Number of residues [c]
PDB, <1.8 Å	11.2 (163.8)	81 (16)
PDB-REDO, <1.8 Å	9.8 (99.9)	81 (16)
PDB, all	24.1 (163.1)	1438 (290)
PDB-REDO, all	29.0 (105.6)	1438 (290)

[a] Only data for the most prevalent carbohydrate residues (NAG, NDG, MAN, BMA, BGC, GLC, GAL, GLA, FUC and FUL) are used.

[b] As reported by *Privateer*

[c] Values for FUC and FUL in parentheses

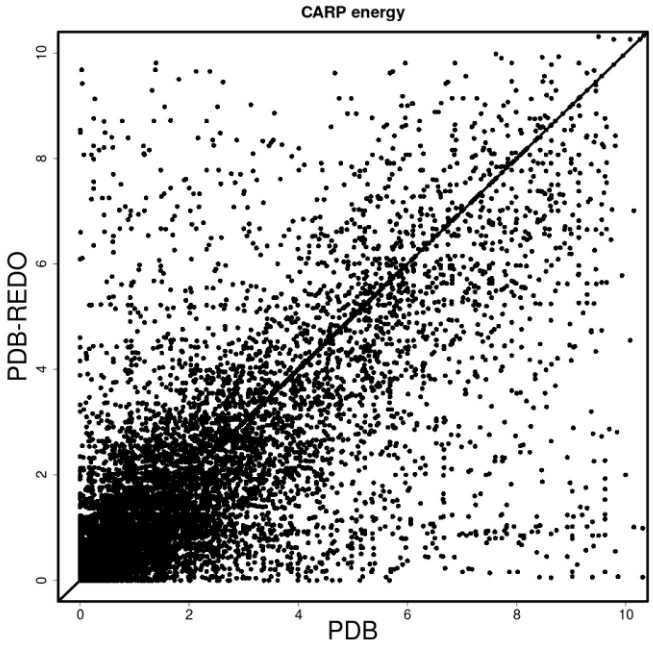

Figure S1 Knowledge-based potentials for glycosidic linkages in the dataset. Potentials are calculated by *CARP* and are given in arbitrary units, lower values are better.

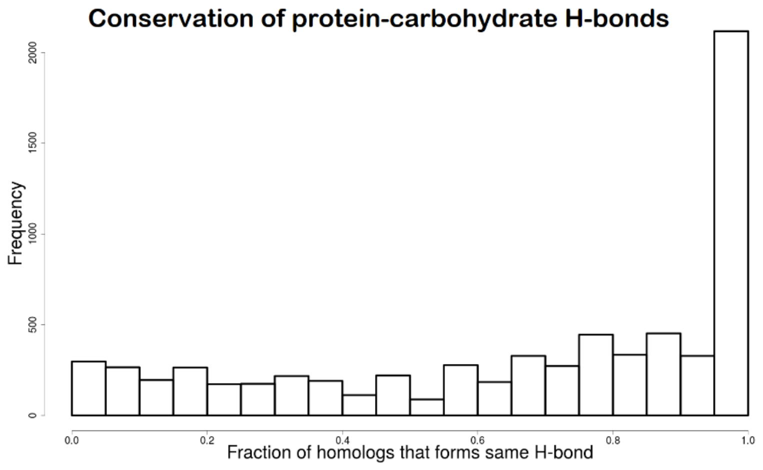

Figure S2 Homologous conservation of protein-carbohydrate H-bonds. Only H-bonds in structures with at least 5 homologous protein chains were considered (6,931). The majority of H-bonds are poorly conserved. Only 2,013 H-bonds could be detected in all available homologs.

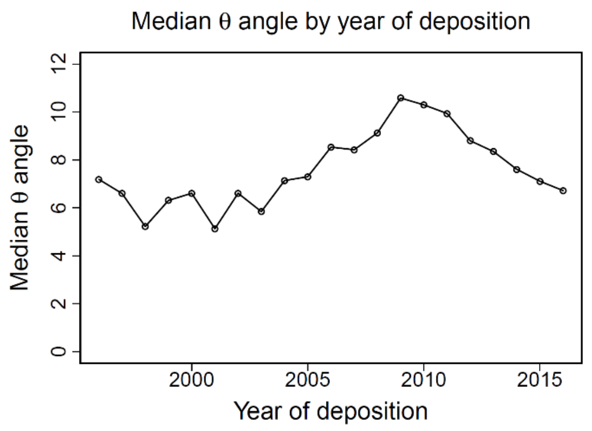

Figure S3 Per-year medians of carbohydrate ring conformation, expressed as θ angle. A downward trend started in 2009 indicating a change of model building and refinement practice.
